# Refractive index gas sensor based on the Tamm state in a one-dimensional photonic crystal: Theoretical optimisation

**DOI:** 10.1038/s41598-020-66427-6

**Published:** 2020-06-16

**Authors:** Zaky A. Zaky, Ashour M. Ahmed, Ahmed S. Shalaby, Arafa H. Aly

**Affiliations:** TH-PPM Group, Physics Department, Faculty of Sciences, Beni-Suef University, Beni-Suef, Egypt

**Keywords:** Nanoscience and technology, Optics and photonics, Physics

## Abstract

Gas sensors are important in many fields such as environmental monitoring, agricultural production, public safety, and medical diagnostics. Herein, Tamm plasmon resonance in a photonic bandgap is used to develop an optical gas sensor with high performance. The structure of the proposed sensor comprises a gas cavity sandwiched between a one-dimensional porous silicon photonic crystal and an Ag layer deposited on a prism. The optimised structure of the proposed sensor achieves ultra-high sensitivity (S = 1.9×10^5^ nm/RIU) and a low detection limit (DL = 1.4×10^−7^ RIU) compared to the existing gas sensor. The brilliant sensing performance and simple design of the proposed structure make our device highly suitable for use as a sensor in a variety of biomedical and industrial applications.

## Introduction

Gas sensing has different applications in many fields such as the food industry, medicine, safety, environment, agriculture, and cosmetic^1,2^. For example, the detection of volatile organic compounds such as acetone and toluene in exhaled breath is used as a biomarker for many diseases^3,4^. In addition, the determination of the concentration of harmful gases such as CO_2_ and N_2_O can be applied as an environmental pollution monitor^5^.

Currently, optical gas sensors are of great interest to researchers because they do not require complicated radioactive/fluorescent labels^6,7^. Surface plasmon resonance, Tamm plasmon (TP) resonance, waveguide, and photonic crystal are all examples of platforms for optical sensing^8–12^.

Photonic crystals (PCs) are useful for a wide range of biomedical and environmental sensing applications. This is due to an impressive set of relevant properties, such as ultrahigh sensitivity, low detection limit, and fast response time^13,14^. PC refers to a range of materials characterised by a periodic refractive index along one, two, or three dimensions (1DPC, 2DPC, or 3DPC, respectively). The propagation of electromagnetic waves in PCs can be controlled because of the photonic bandgap (PBG)^15–17^. 1DPCs are more appropriate for most applications, given their low cost and ease of fabrication compared to 2DPCs and 3DPCs^18^.

Recently, PCs have been widely used in various sensor systems. A high-precision gas index sensor, which was proposed by Jágerská *et al*., reached a sensitivity of 510 nm/RIU based on a PC air-slot cavity^19^. Hua-Jun studied a surface plasmon resonance nanocavity antenna array for use as a gas sensor with a high sensitivity of 3200 nm/RIU^20^. Wang *et al*. suggested a guided-mode resonance gas sensor with a sensitivity as high as 748 nm/RIU^21^. Pevec and Donlagic designed a fiber-optic Fabry-Perot gas sensor with a sensitivity of 1550 nm/RIU^22^. García-Rupérez *et al*. presented a highly sensitive device for antibody detection using the slow light regime of a PC waveguide^23^. Chen *et al*. designed a PC/Ag/graphene structure to function as a refractive index sensor based on the Tamm state, with a numerical sensitivity of 1178.6 nm/RIU^24^. Auguié *et al*. studied TP resonance at the interface between a metal/mesoporous PC. The numerical results showed that the sensitivity was approximately 55 nm/RIU^25^.

Recently, 1DPCs based on multilayers of porous silicon (PSi) have become an effective solution for the design of novel biosensors^26–28^. PSi characteristically provides high surface area and low mass within small volumes. The optical properties of PSi can be controlled by changing the size of the pores and/or material that fills the pores^27^. Moreover, PSi is compatible with integrated electronic circuits.

In contrast, TP resonance is used as an optical sensor technology with high performance. TPs can be created inside the bandgap by adding a metal layer in front of the 1DPC^25^. The wavelength location of the TP resonant dip can be shifted by changing the effective refractive index of the 1DPC structure or surrounding medium. This is the principle used in the proposed structure for detecting small changes in the refractive index of the gas.

The present work aims to introduce a high-performance gas sensor based on an effective combination of the TP features and novel properties of PSi photonic crystals.

## Sensor Design

The proposed sensor consists of a one-dimensional porous silicon photonic crystal (PSi-1DPC) covered with an Ag layer. In addition, there is a cavity layer between the two materials for the gas to be detected. The Ag layer is deposited on a prism of glass (n_0_ = 1.5)^29–31^.

**Figure 1:**
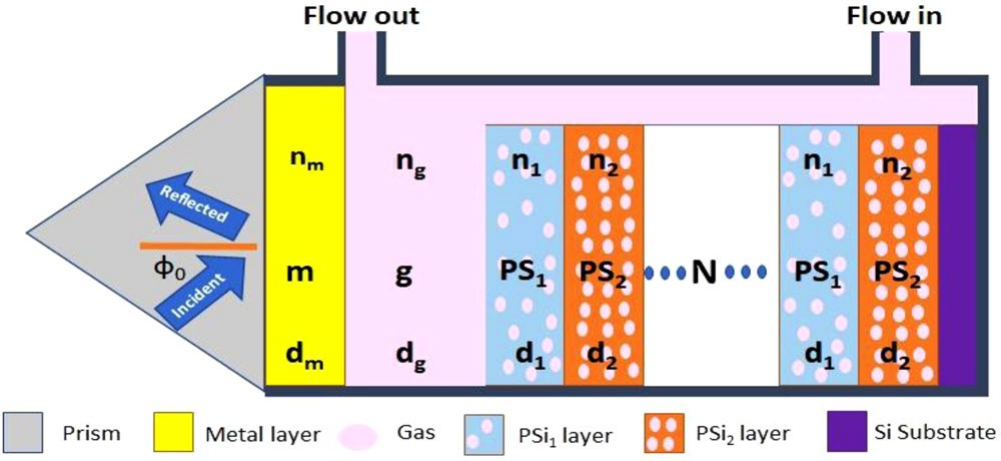
Schematic of the proposed biosensor consisting of prism/Ag/gas/(PSi_1_/PSi_2_)^N^/Si.

Figure 1 shows a schematic representation of the proposed structure, which is considered as prism/Ag/gas/(PSi_1_/PSi_2_)^N^/Si, where gas, PSi_1_, and PSi_2_ refer to the gas cavity and the first and second PSi layers, respectively. N indicates the period number. The silicon layer acts as a substrate for the sensor.

The gas sample is injected into the inlet, which is in contact with the upper surface of the structure. This allows the gas to fill the cavity layer and pores of PSi, as shown in Fig. 1.

The refractive index of Ag was obtained from the Drude model^32–34^:

1 $${{\rm{n}}}_{{\rm{m}}}=\sqrt{1-{{\rm{\omega }}}_{{\rm{p}}}^{2}/({{\rm{\omega }}}^{2}+{\rm{i}}\,{\rm{\gamma }}\,{\rm{\omega }})},$$

where the plasma frequency and damping factor are represented by ω_p_ = 2.18 PHz and γ = 4.353 THz, respectively^35^. Ag was selected because it has a relatively low imaginary part of the dielectric constant (low absorption loss) compared to gold, platinum, and copper^36^.

The refractive index of silicon (n_Si_) is given by^37^:

2 $${{\rm{n}}}_{{\rm{Si}}}=\sqrt{\left(1+\frac{10.6684293\,{{\rm{\lambda }}}^{2}}{{{\rm{\lambda }}}^{2}-{0.301516485}^{2}}+\frac{0.0030434748\,{{\rm{\lambda }}}^{2}}{{{\rm{\lambda }}}^{2}-{1.13475115}^{2}}+\frac{1.54133408\,{{\rm{\lambda }}}^{2}}{{{\rm{\lambda }}}^{2}-{1104}^{2}}\right)},$$

where λ is the wavelength (μm). The porosities of the PSi_1_ and PSi_2_ layers are 32% and 74%, respectively, based on the results of a previous experimental work^38^.

Multilayer PSi-1DPC can be prepared by electrochemical etching of a silicon wafer using hydrogen fluoride as the electrolyte^28,38–41^.

## Theoretical Model

The transfer matrix method (TMM) is used to study the interaction between the incident electromagnetic (EM) waves (S-polarized) and the proposed structure. The details of TMM can be found in many articles^42–46^. The following matrices describe the proposed structure:

3 $$\begin{array}{l}{\rm{H}}=({{\rm{h}}}_{{\rm{m}}})({{\rm{h}}}_{{\rm{gas}}}){({{\rm{h}}}_{1}{{\rm{h}}}_{2})}^{{\rm{N}}}=(\begin{array}{ll}{{\rm{H}}}_{11} & {{\rm{H}}}_{12}\\ {{\rm{H}}}_{21} & {{\rm{H}}}_{22}\end{array})\end{array}$$

where h_1_, h_2_, h_gas_ and h_m_ are the characteristic matrix for PSi_1_, PSi_2_, the gas sample, and the metallic layer. H_11_, H_12_, H_21_, and H_22_ are the transfer matrix elements for the total structure. The following equation gives the reflection coefficient:

4 $${\rm{r}}=\frac{({{\rm{H}}}_{11}+{{\rm{H}}}_{12}\,{{\rm{p}}}_{{\rm{s}}}){{\rm{p}}}_{0}-({{\rm{H}}}_{21}+{{\rm{H}}}_{22}\,{{\rm{p}}}_{{\rm{s}}})}{({{\rm{H}}}_{11}+{{\rm{H}}}_{12}\,{{\rm{p}}}_{{\rm{s}}}){{\rm{p}}}_{0}+({{\rm{H}}}_{21}+{{\rm{H}}}_{22}\,{{\rm{p}}}_{{\rm{s}}})}$$

Where p_0_ = n_0_ cos φ_0_ (for prism) and p_s_ = n_s_ cos φ_s_ (for Si substrate). Also, φ_0_ indicates the incident angle of the electromagnetic waves from the prism to the structure.

Finally, the reflectance of the proposed structure is given by:

5 $${\rm{R}}( \% )=100{\rm{x}}|{{\rm{r}}}^{2}|$$

## Results and Discussions

In this section, we calculate the refractive index of the PSi (n_Psi_). Next, we study how the performance of our sensor is affected by changes in the gas refractive index, number of periods, metallic layer thickness, refractive index of the prism, layer thickness of the gas, and angle of incidence of the electromagnetic waves.

### Refractive index of porous silicon ${{\bf{n}}}_{{\bf{P}}{\bf{s}}{\bf{i}}}$

**Figure 2:**
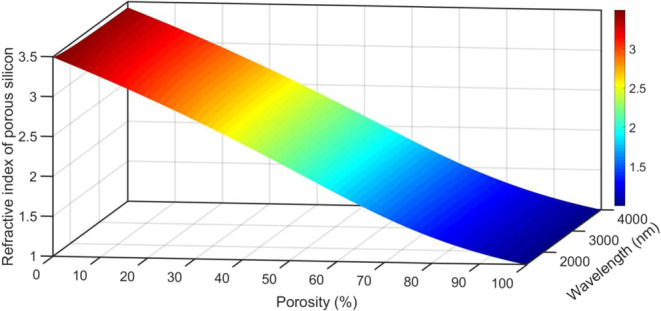
Changes in the n_Psi_ layer as a function of wavelength and porosity.

The index of refraction of the PSi layer (n_Psi_) filled with gas can be obtained by applying Bruggeman’s effective medium approximation^26,47,48^:

6 $$\begin{array}{c}{{\rm{n}}}_{{\rm{Psi}}}=0.5\sqrt{ {\mathcal M} +\sqrt{{ {\mathcal M} }^{2}+8{{\rm{n}}}_{{\rm{si}}}^{2}{{\rm{n}}}_{{\rm{gas}}}^{2}}}\\  {\mathcal M} =3{\rm{P}}({{\rm{n}}}_{{\rm{gas}}}^{2}-{{\rm{n}}}_{{\rm{si}}}^{2})+(2\,{{\rm{n}}}_{{\rm{si}}}^{2}-{{\rm{n}}}_{{\rm{gas}}}^{2}),\end{array}$$

where n_Psi_, n_si_, and n_gas_ are the refractive indices of the PSi layer, silicon, and gas inside the pores, respectively. The refractive index of the PSi layer decreases from 3.50 to 1.00026 as the ratio of the porosity of silicon filled with a gas of refractive index 1.00026 changes from 0% to 100% (Fig. 2).

### Reflectance spectra for prism/PSi-1DPC and prism/Ag/gas/PSi-1DPC

In all calculations of the reflectance spectra of the electromagnetic waves, the PSi_1_ and PSi_2_ layers have thicknesses of d_1_ = 200 nm and d_2_ = 600 nm with porosities of 32% and 74%, respectively.

**Figure 3:**
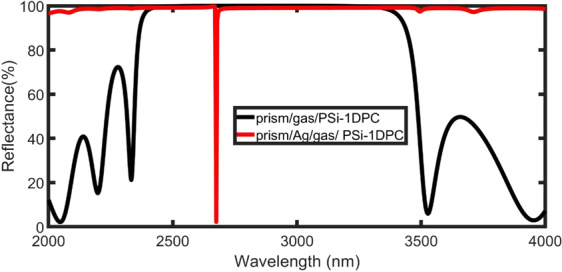
Reflectance for ${\rm{prism}}/{\rm{gas}}/\,$PSi-1DPC and ${\rm{prism}}/\,$Ag/gas/ PSi-1DPC as a function of the wavelength with a normal incident angle, N = 8, d_gas_ = 4000 nm, n_gas_ = 1.00026, and d_m_ = 30 nm.

Figure 3 shows the reflectance of the ${\rm{prism}}/{({{\rm{PSi}}}_{1}/{{\rm{PSi}}}_{2})}^{8}/{\rm{Si}}$ as a function of the wavelength (black curve). The gas inside the pore has a refractive index of n_gas_ = 1.00026. The number of periods is eight (N = 8) and light is normally incident on the structure (φ_0_ = 0°). As illustrated by this figure, there is a wide PBG (high reflection) owing to the high refractive index contrast between the two layers, PSi_1_ and PSi_2_. This PBG results from the constructive interference of the reflected waves at the interface between different layers. Outside the PBG, ripples appear in the reflectance spectrum with high reflectance.

For the ${\rm{prism}}/\,$ ${\rm{A}}{\rm{g}}/{\rm{g}}{\rm{a}}{\rm{s}}/{({{\rm{P}}{\rm{S}}{\rm{i}}}_{1}/{{\rm{P}}{\rm{S}}{\rm{i}}}_{2})}^{8}/{\rm{S}}{\rm{i}}$ structure, the layer of the gas cavity and Ag have thicknesses of d_gas_ = 4000 nm and d_m_ = 30 nm, respectively. In this case, the PBG expands, and the ripples outside the bandgap almost disappear (red curve in Fig. 3). In addition, a Tamm resonant dip appears with ${{\rm{\lambda }}}_{{\rm{T}}}=2675\,{\rm{nm}}$ inside the PBG as a result of the electromagnetic waves confined between the Ag layer and distributed Bragg reflector^26,49,50^.

### Effect of small changes in the gas refractive index

**Figure 4:**
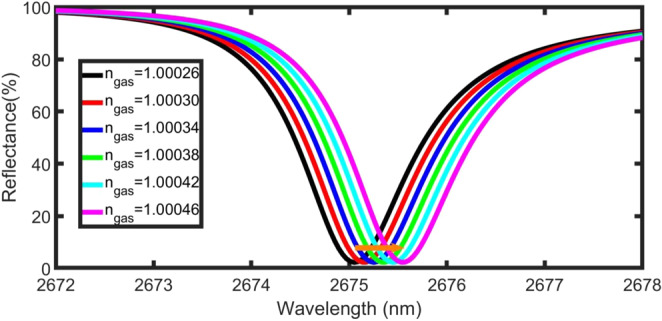
Reflectance spectra of the proposed sensor as a function of wavelength and gas refractive index at d_gas_ = 4000 nm, d_m_ = 30 nm, N = 8, and φ_0_ = 0°.

Figure 4 shows the dip position of the TP resonance for the ${\rm{prism}}/{\rm{Ag}}/{\rm{gas}}/{({{\rm{PSi}}}_{1}/{{\rm{PSi}}}_{2})}^{8}/{\rm{Si}}$ structure at different gas refractive indices. All parameters were maintained as in the previous case (d_1_ = 200 nm, d_2_ = 600 nm, d_gas_ = 4000 nm, d_m_ = 30 nm, N = 8, and φ_0_ = 0°). The refractive index of the gas sample (n_gas_) changes from 1.00026 to 1.00046 (Δn_gas_ = 2 × 10^−4^).

Increasing the refractive index of the gas inside the pores causes an increase in the effective refractive index of the PSi layers. Consequently, the effective refractive index of the ${\rm{prism}}/\,$Ag/gas/PSi-1DPC structure increases. This leads to a TP resonance shift to longer wavelengths (red-shift)^34,51^, in accordance with Bragg’s law.

The sensitivity (S) is the most important parameter used to characterise the performance of a sensor. It is calculated through the following equation:

7 $${\rm{S}}=\frac{{\Delta {\rm{\lambda }}}_{{\rm{T}}}}{\Delta {\rm{n}}}=\frac{{{\rm{\lambda }}}_{{\rm{T}}}^{1.00046}-{{\rm{\lambda }}}_{{\rm{T}}}^{1.00026}}{1.00046-1.00026}=\frac{{{\rm{\lambda }}}_{{\rm{T}}}^{1.00046}-{{\rm{\lambda }}}_{{\rm{T}}}^{1.00026}}{0.0002},$$

where ${{\rm{\lambda }}}_{{\rm{T}}}$ is the position of the Tamm resonance dip. By increasing the gas refractive index from 1.00026 to 1.00046, the TP resonance dip is shifted from ${{\rm{\lambda }}}_{{\rm{T}}}$ = 2675.16 to 2675.68 nm, as seen in Fig. 4. The sensitivity in these conditions is approximately 2600 nm/RIU.

To achieve the highest performance, different parameters of the proposed sensor, such as the number of periods, metallic layer thickness, prism refractive index, gas layer thickness, and incident angle were optimised.

### Effect of number of periods

**Figure 5:**
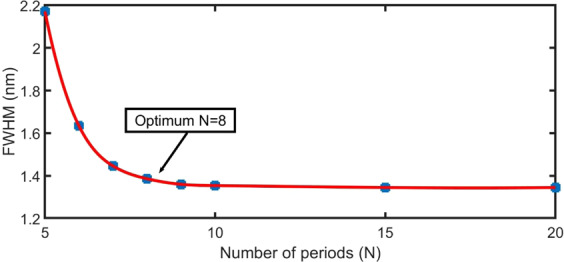
Variation in the FWHM as a function of the number of unit cells N at d_m_ = 30 nm, n_gas_ = 1.00026, n_prism_ = 1.5, φ_0_ = 0°, and d_gas_ = 4000 nm.

By increasing the number of periods, the sensitivity does not change (S = 2600 nm/RIU). In addition to the sensitivity, the study of the full width at half maximum (FWHM) of the resonance dip is another significant parameter for the performance of the sensor. A high-performance sensor should have a narrow resonant dip to achieve high resolution^52^. Figure 5 shows the behaviour of the FWHM as a function of the number of unit cells (${\rm{N}}$), at Ag layer thickness d_m_ = 30 nm, gas layer thickness d_gas_ = 4000 nm, n_gas_ = 1.00026, n_prism_ = 1.5, and normal incidence of electromagnetic waves.

The FWHM value decreases (from 2.17 to 1.39 nm) with an increase in the number of periods from N = 5 to 8. Above N = 8, the FWHM value seems to be constant^25^. Therefore, N = 8 is considered as the optimum number of layers for the next calculation.

### Effect of Ag layer thickness

**Figure 6:**
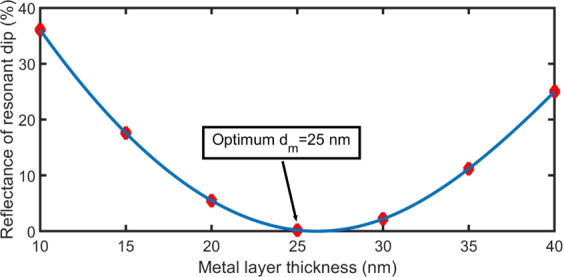
Variation of the reflectance of the resonant dip as a function of the metallic layer thickness at n_gas_ = 1.00026, φ_0_ = 0°, d_gas_ = 4000 nm, n_prism_ = 1.5, and N = 8.

Figure 6 shows the variation in the reflectance of the Tamm resonant dip (R_T_) as a function of the Ag layer thickness at n_gas_ = 1.00026, φ_0_ = 0°, d_gas_ = 4000 nm, n_prism_ = 1.5, and N = 8.

When the Ag layer has a thickness of 25 nm, the reflectance of the resonant dip decreases to zero, and the optical energy is consumed by absorption^25^. Hence, strong confined electromagnetic waves occur between the Ag and ${\rm{gas}}/{({{\rm{PSi}}}_{1}/{{\rm{PSi}}}_{2})}^{8}/{\rm{Si}}$ structure^26,49,50^, which is crucial for sensing applications. Therefore, d_m_ =25 nm is considered as the optimum thickness for the Ag layer, because it achieves zero reflectance. When the thickness of the Ag layer differs from the optimal value, the reflectance of the resonant dip increases resulting in the low coupling of the TP, as seen in Fig. 6. This behaviour is similar to the results observed in a previous study^53^.

### Effect of prism refractive index

To study the effect of the refractive index of the prism on the reflectance of the structure, we changed the refractive index of the prism from 1.4 to 2.5^54,55^. According to the principle of total internal reflection, the critical angle (φ_c_) depends on the values of the refractive index of the prism and gas for the ${\rm{prism}}/\,$gas/PSi-1DPC structure. The critical angle can be calculated using the following equation:

8 $${{\rm{\varphi }}}_{c}={\sin }^{-1}\left(\frac{{{\rm{n}}}_{{\rm{gas}}}}{{{\rm{n}}}_{{\rm{prism}}}}\right)$$

**Figure 7:**
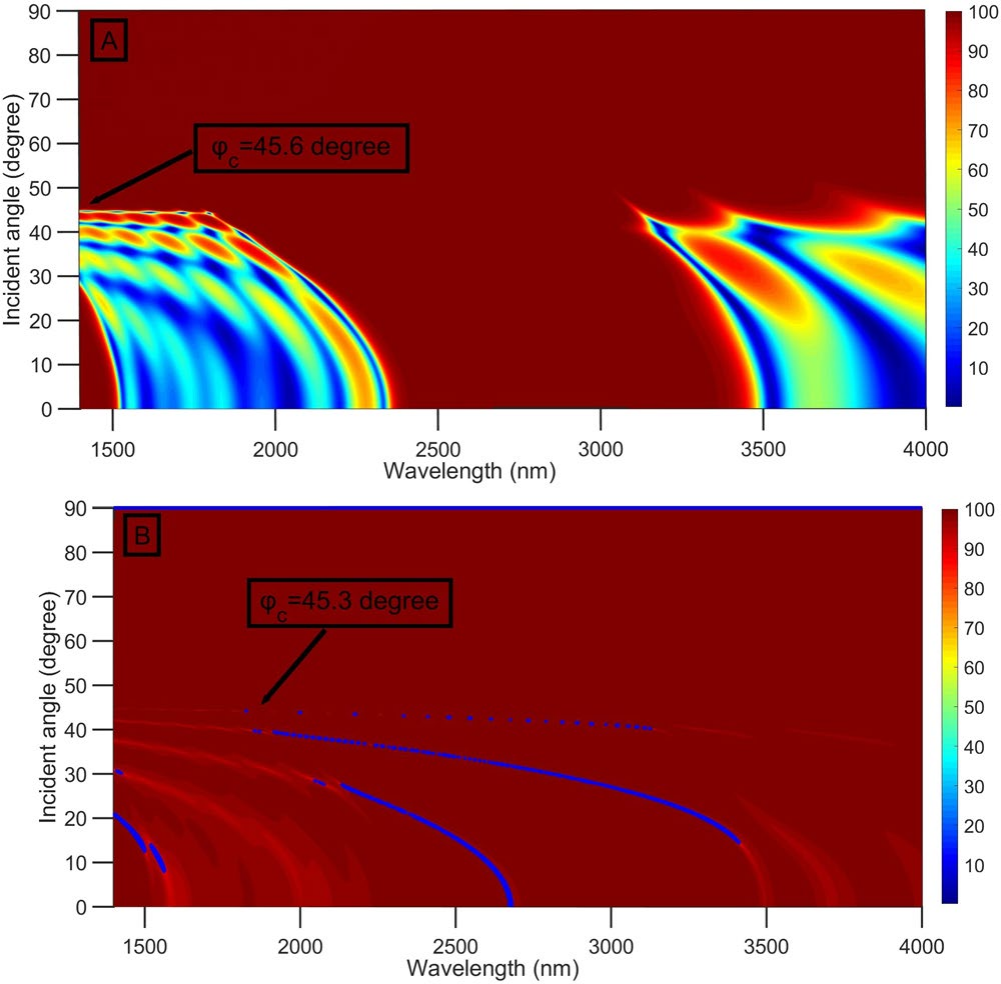
Reflectance spectra as a function of wavelength and incident angle at n_gas_ = 1.00026, d_gas_ = 4000 nm, d_m_ = 25 nm, ${{\rm{n}}}_{{\rm{prism}}}=1.4$ and N = 8 for (**A**) ${\rm{prism}}/{\rm{gas}}/\,$PSi-1DPC and (**B**) ${\rm{prism}}/{\rm{Ag}}/{\rm{gas}}/\,$PSi-1DPC.

By taking ${{\rm{n}}}_{{\rm{gas}}}=1.00026$, ${{\rm{d}}}_{{\rm{m}}}=0$, and ${{\rm{n}}}_{{\rm{prism}}}=1.4$, the value of ${{\rm{\varphi }}}_{c}$ equals 45.6°, as clearly shown in Fig. 7A. When ${n}_{{\rm{prism}}}\,$increases from 1.4 to 2.5°, the critical angle decreases from 45.6 to 23.6°. Above the critical angle, a total reflection occurs without the appearance of any resonant dips for the ${\rm{prism}}/{\rm{gas}}/\,$PSi-1DPC (Fig. 7A) and ${\rm{prism}}/{\rm{Ag}}/{\rm{gas}}/\,$PSi-1DPC (Fig. 7B) structures at ${{\rm{n}}}_{{\rm{prism}}}=1.4$. The optimum value of ${{\rm{n}}}_{{\rm{prism}}}$ is 1.4, which achieves a high critical angle, and hence a wide range of angles will be studied in the next section.

### Effect of gas layer thickness

An increasing gas layer thickness leads to an increase in the volume fraction (ϕ) of the gas layer and geometric path of the electromagnetic wave inside the 1DPC. Therefore, the interaction between the electromagnetic and gas molecules is enhanced. Hence, the proposed structure will be more sensitive to the changes in the refractive index of the gas by increasing the thickness of the gas layer.

**Figure 8:**
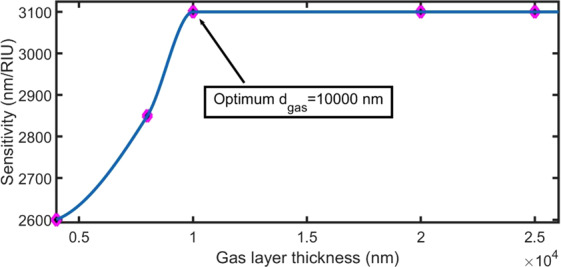
Effect of the gas layer thickness on sensitivity at N = 8, d_m_ = 25 nm, n_prism_ = 1.4, and ${\varphi }_{0}$ = 0°.

As the gas layer thickness increases from 4000 nm to 10000 nm, the sensitivity increases rapidly from 2600 nm/RIU to 3100 nm/RIU. The sensitivity is not affected when the gas layer thickness is increased to more than 10000 nm, as seen in Fig. 8. This result agrees with previous theoretical and experimental studies^14,56^. A thickness of 10000 nm will be considered as the optimum thickness of the gas layer and used for the subsequent simulations.

### Effect of incident angle

The increase in the incident angle of the S-polarised electromagnetic wave causes a blue-shift (short wavelength) to the Tamm resonance dip, according to the Bragg–Snell law:

9 $${\rm{k}}\,{\rm{\lambda }}=2\,{\rm{D}}\sqrt{{{\rm{n}}}_{{\rm{eff}}}^{2}-{\sin }^{2}\,{{\rm{\varphi }}}_{0}},$$

where k is the order of diffraction, λ is the wavelength, D is the interplanar spacing, n_eff_ is the effective refractive index, and φ_0_ is the angle of incidence.

**Figure 9:**
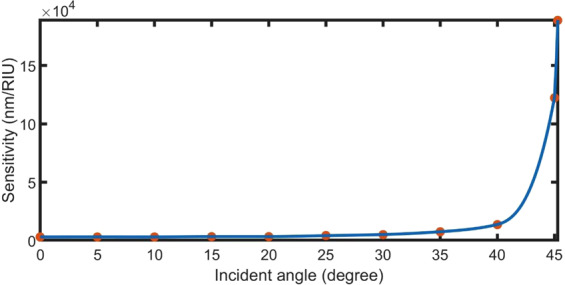
Effect of the incident angle on sensitivity at N = 8, d_3_ = 10000 nm, n_prism_ = 1.4, and d_m_ = 25 nm.

When the angle of incidence of the electromagnetic waves increases, the wave travels a long geometric path through the gas layer^57^. Therefore, the interactions between the electromagnetic waves and gas molecules are improved. Hence, the sensitivity of the proposed structure is enhanced by increasing the incident angle. By increasing the incident angle from 0 to 45.30^o^ (below ${{\rm{\varphi }}}_{c}$), the sensitivity value increased from 3100 to 188750 nm/RIU, as shown in Fig. 9. The increase in sensitivity at angles higher than 40° in Fig. 9 is due to the jump in the geometric path of the wave with the increase of the incident angle.

### Sensor analysis under optimum conditions

**Figure 10:**
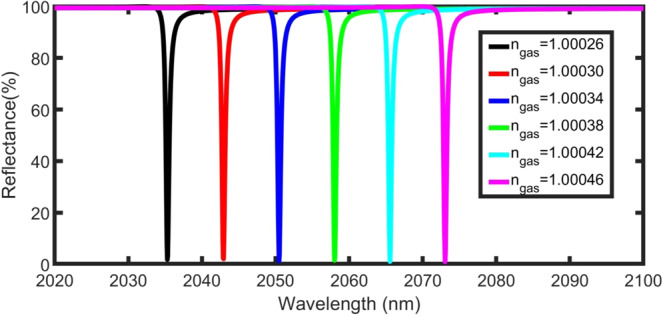
Reflectance spectra of the proposed sensor under optimum conditions with different gas refractive indices.

From the above results, the optimum conditions are N = 8, d_m_ = 25 nm, d_gas_ = 10000 nm, n_prism_ = 1.4, and φ_0_ = 45.3°. Figure 10 shows the reflectance spectra of the proposed sensor with different gas refractive indices under these optimum conditions. Increasing the gas refractive index leads to shifting the position of the TP mode towards longer wavelengths.

**Figure 11:**
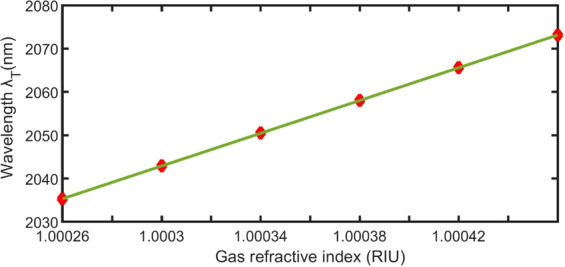
Linear relation between the refractive index of the gas and TP dip positions.

Figure 11 presents a linear fitting of the refractive index of the gas and its dip positions. The slope of the linear fitting refers to the average sensitivity (1.9×10^5^ nm/RIU) of our sensor according to the following equation:

10 $${{\rm{\lambda }}}_{{\rm{T}}}=190000\,{{\rm{n}}}_{{\rm{gas}}}-187000,\,({{\rm{R}}}^{2}=0.999)$$

The quality factor (QF), figure of merit (FoM), and detection limit (DL) are usually used to characterise the efficiency and performance of the sensor. An excellent sensor has high QF values that demonstrate the ability of the sensor to have a narrow bandwidth^52^. The QF can be calculated according to the following equation:

11 $${\rm{QF}}=\frac{{{\rm{\lambda }}}_{{\rm{T}}}}{{\rm{FWHM}}}$$

The ratio between the S and the FWHM is referred to as FoM, which is obtained by

12 $${\rm{FoM}}=\frac{{\rm{S}}}{{\rm{FWHM}}}$$

The DL is inversely proportional to ${\rm{S}}$ and QF according to^58^:

13 $${\rm{DL}}=\frac{{{\rm{\lambda }}}_{{\rm{T}}}}{20\,{\rm{S}}\,{\rm{QF}}}$$

Table 1 illustrates that the FoM of our sensor is 3.6×10^5^ RIU and the QF is 4×10^3^. The high value of the FoM indicates that the proposed sensor has high sensitivity and narrow FWHM simultaneously. In addition, the DL of the sensor is approximately 1.4×10^−7^ RIU, which indicates the smallest detectable refractive index change.

**Table 1:** Different parameters of the proposed sensor at optimum conditions.

n (RIU)	${{\rm{\lambda }}}_{{\rm{T}}}$ (nm)	S (nm/RIU)	FWHM (nm)	FoM ×10^5^ (/RIU)	QF ×10^3^
1.00026	2035.3	------	0.52	------	4
1.00030	2042.9	190 000	0.52	3.6	4
1.00034	2050.5	190 000	0.52	3.6	4
1.00038	2058.0	187 500	0.52	3.6	4
1.00042	2065.6	190 000	0.52	3.6	4
1.00046	2073.1	187 500	0.52	3.6	4

Table 2 compares the values of S, FoM, and DL for the present study with a number of previous experimental and theoretical works^24,59–63^ and highlights how the sensitivity of the proposed sensor is higher. Thus, the proposed device with much better performance than hitherto experimentally demonstrated can be constructed^59,60^.

**Table 2:** Comparison of sensitivity, figure of merit, and detection limit values of the present work with some experimental and theoretical previous works.

Year	S (nm / RIU)	FoM (/RIU)	DL (RIU)
2015^61^	970	12.76 × 10^−3^	–
2016^62^	450	~800	~1.6 × 10^−4^
2016^24^	1179	2.2 × 10^3^	~2.2 × 10^−5^
2017^59^	850	–	–
2019^60^	<70	1.04	–
2020^63^	9615	–	–
**This work**	**1.9** × **10**^**5**^	**3.6** × **10**^**5**^	**1.4** × **10**^**−7**^

## Conclusion

We presented a model for a possible large sensitivity gas sensor based on the Tamm state in a one-dimensional photonic crystal. The number of layers, metallic cap layer thickness, prism refractive index, gas layer thickness, and incident angle of the electromagnetic wave were optimised to achieve a biosensor with high performance. The proposed sensor has S = 1.9 × 10^5^ nm/RIU, FoM = 3.6 × 10^5^ RIU, QF = 4 × 10^3^, and DL = 1.4 × 10^−7^ RIU. The results obtained in the present work motivated us to plan future work focused on the design of photonic crystals as gas sensors for biomedical applications, in particular for the analysis of different gases in the human breath.
